# Automated approach for segmenting gross tumor volumes for lung cancer stereotactic body radiation therapy using CT-based dense V-networks

**DOI:** 10.1093/jrr/rraa132

**Published:** 2021-01-22

**Authors:** Yunhao Cui, Hidetaka Arimura, Risa Nakano, Tadamasa Yoshitake, Yoshiyuki Shioyama, Hidetake Yabuuchi

**Affiliations:** Department of Health Sciences, Graduate School of Medical Sciences, Kyushu University, 3-1-1, Maidashi, Higashi-ku, Fukuoka 812-8582, Japan; Department of Health Sciences, Faculty of Medical Sciences, Kyushu University, 3-1-1, Maidashi, Higashi-ku, Fukuoka 812-8582, Japan; Department of Health Sciences, Graduate School of Medical Sciences, Kyushu University, 3-1-1, Maidashi, Higashi-ku, Fukuoka 812-8582, Japan; Department of Clinical Radiology, Graduate School of Medical Sciences, Kyushu University, 3-1-1, Maidashi, Higashi-ku, Fukuoka 812-8582, Japan; Saga International Heavy Ion Cancer Treatment Foundation, 3049 Harakogamachi, Tosu-shi, Saga 841-0071, Japan; Department of Health Sciences, Faculty of Medical Sciences, Kyushu University, 3-1-1, Maidashi, Higashi-ku, Fukuoka 812-8582, Japan

**Keywords:** deep learning, segmentation, dense V-networks, lung stereotactic body radiation therapy

## Abstract

The aim of this study was to develop an automated segmentation approach for small gross tumor volumes (GTVs) in 3D planning computed tomography (CT) images using dense V-networks (DVNs) that offer more advantages in segmenting smaller structures than conventional V-networks. Regions of interest (ROI) with dimensions of 50 × 50 × 6–72 pixels in the planning CT images were cropped based on the GTV centroids when applying stereotactic body radiotherapy (SBRT) to patients. Segmentation accuracy of GTV contours for 192 lung cancer patients [with the following tumor types: 118 solid, 53 part-solid types and 21 pure ground-glass opacity (pure GGO)], who underwent SBRT, were evaluated based on a 10-fold cross-validation test using Dice’s similarity coefficient (DSC) and Hausdorff distance (HD). For each case, 11 segmented GTVs consisting of three single outputs, four logical AND outputs, and four logical OR outputs from combinations of two or three outputs from DVNs were obtained by three runs with different initial weights. The AND output (combination of three outputs) achieved the highest values of average 3D-DSC (0.832 ± 0.074) and HD (4.57 ± 2.44 mm). The average 3D DSCs from the AND output for solid, part-solid and pure GGO types were 0.838 ± 0.074, 0.822 ± 0.078 and 0.819 ± 0.059, respectively. This study suggests that the proposed approach could be useful in segmenting GTVs for planning lung cancer SBRT.

## INTRODUCTION

Lung cancer, the most common fatal malignancy in the developed world [1], causes >1.3 million deaths worldwide each year, according to the world health organization (WHO) [2–3]. It is the most commonly occurring malignant cancer in men and the third most commonly occurring cancer in women [4–5]. Despite the development of multi-modality treatments over the past decade, lung cancer remains the leading cause of death from malignant cancers, accounting for ~25% of all cancer deaths [6]. The treatment of lung cancer should be selected appropriately according to the clinical stage identified by scientific evidence [7]. In general, there are four main treatments for lung cancer, namely surgery, radiation therapy, chemotherapy and immunotherapy. Stereotactic body radiotherapy (SBRT) refers to one of the treatment options for patients in the early stages (I or II) of non-small cell lung cancer (NSCLC) and who are medically inoperable or refuse surgery. Compared to conventional radiotherapy, SBRT can deliver higher doses (12, 20, 22 Gy/fraction and more) to small targets ($\le$50 mm) over 3–5 treatment fractions, using multiple conformal coplanar and non-coplanar beams [8–9]. Therefore, the contours of gross tumor volumes (GTVs), which are analyzed to produce estimates of clinical target volumes (CTVs) and planning target volumes (PTVs), should be as accurate as possible [10–11]. However, the GTV regions are manually delineated from treatment planning computed tomography (CT) images by treatment planners (e.g. radiation oncologists) with differing levels of experience and skills [12–15]. Thus, manual delineations can cause intra- and inter-observer variabilities in GTV contours, making them less repeatable and reproducible, leading to variability in treatment dose distributions. To address these issues, automated segmentation approaches are in high demanded in clinical SBRT practices for reducing observer variabilities.

A number of efforts have been made to develop more reproducible and more accurate GTV segmentation approaches using machine learning techniques such as deep learning in various imaging modalities (e.g. CT, positron emission tomography (PET), and magnetic resonance imaging) [16–23]. Automated approaches for segmenting GTV regions have been reported based on machine learning using PET- or PET/CT-based images [16–17]. Moreover, ^18^F-fluorodeoxyglucose (FDG)-PET directly shows relevant biomedical information, indicating the potential to improve the accuracy and achieve a more stable result of target volume delineation [13, 24]. If artificial intelligence, including machine learning or deep learning, is assumed to have the same ability to segment lung tumors for SBRT as a radiation oncologist with >6 years of experience, there would be some benefits for radiation oncologists, i.e. reducing the contour variability, and their time and labor for delineation of tumor contours.

Zhong *et al.* [25] studied deep-learning co-segmentation models of GTV regions in PET-CT images with 38 and 22 sets of training and test datasets, respectively. The models were based on deep fully convolutional networks (DFCN), which consisted of two coupled 3D U-nets [26] with an encoder–decoder architecture in lung SBRT. Additionally, Zhao *et al.* [27] studied deep-learning co-segmentation in PET/CT images with 48 and 36 sets of training and validation datasets, respectively, or segmentation in a single modality (PET or CT only) using multi-modality, fully convolutional neural networks (FCNs) in lung cancer. In contrast to past studies with machine learning [16, 17], deep learning techniques can automatically generate image features and segment the GTV regions of lung cancer.

However, there are three issues in the past studies. The use of the PET images has an issue of motion artifacts (e.g. blurring). Although using PET images may increase the performance of segmentation, it may also include ambiguous information in the datasets because of motion artifacts. Motion artifacts may cause non-negligible misregistration on tumor boundaries between PET and CT images. Reducing them in PET images is difficult due to the long duration of PET scans, which are taken under free respiration [28]. Furthermore, the number of PET scanners including PET/CT scanners is limited to <0.5 per 100 000 population compared with >8 CT scanners per 100 000 population in Japan [29]. Another benefit of using only CT images is that reducing PET examinations may not cause additional costs and radiation exposures to patients. Therefore, to diminish the risk of misregistration and extra costs, it is more appropriate to make use of only CT images for segmenting GTV regions, rather than using both PET and CT images. The number of cases investigated in previous deep learning-based studies [25, 27] is insufficient. Finally, the past studies did not employ the latest deep learning architectures and methods, like dense V-networks (DVNs) [30], which have the advantage of being able to segment smaller structures for small lung cancer in SBRT. Hence, our aim in this study was to develop an automated segmentation approach for small GTVs in lung cancer SBRT using CT-based densely connected V-Net. This approach has more advantages than conventional V-networks [31] in the segmentation of smaller structures.

## MATERIALS AND METHODS

**Fig. 1.**
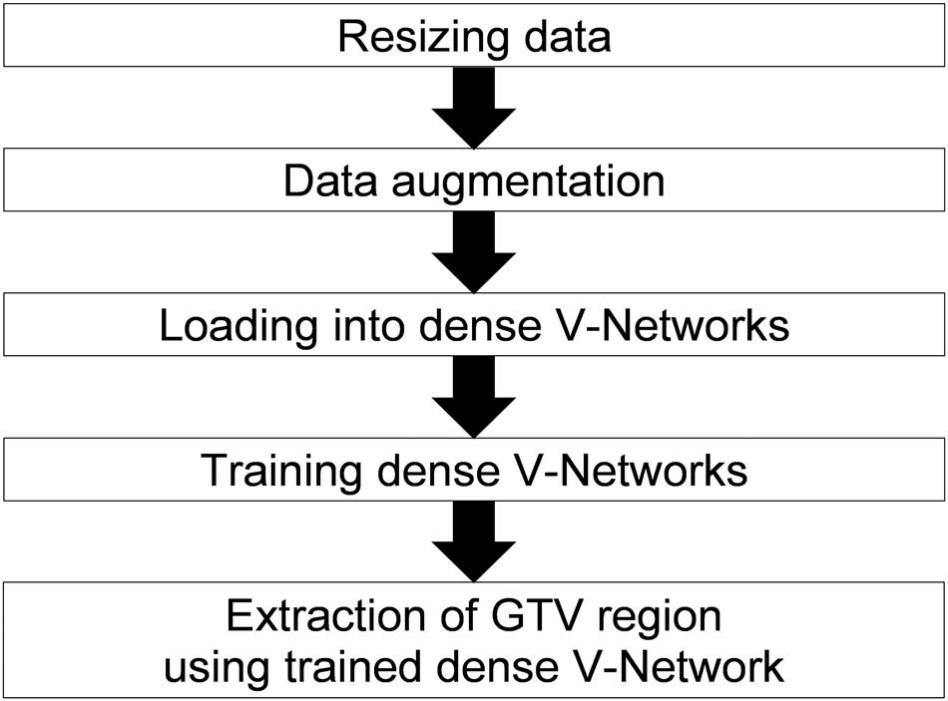
Overall scheme of the automated approach for segmenting GTV regions.

Figure 1 shows an overall scheme of the proposed approach for segmenting GTV regions. The procedure for the proposed approach is explained in the next section. An open source convolutional network platform [32] was applied to the CT-based DVNs to solve the GTV segmentation problem.

### Clinical cases

This study was performed under the approval of an institutional review board of our university hospital. We selected 192 NSCLC patients (40–92 years old; mean age: 74 years; 70 females; 122 males; mean effective diameter of GTV: 11.039 mm; effective diameter range: 3.462–24.716 mm) who received SBRT at the university hospital. The patients were free to breathe when taking the planning CT images. The planning CT images were acquired from a 4-slice CT scanner (Mx 8000; Philips, Amsterdam, The Netherlands) with a matrix size from 512 × 512 × 103 to 512 × 512 × 235 pixels; pixel sizes of 0.781, 0.879 and 0.977 mm; and slice thicknesses of 2 or 3.2 mm. For the size of CT images used in this research, the first two values are the number of pixels in *x* and *y* directions, and the third value is the number of slices (*z* direction). The GTV contours were delineated on planning CT images using a commercially available radiation treatment planning (RTP) system (Eclipse version 6.5 or 8.1; Varian Medical System Inc., Palo Alto, USA), based on consensuses between two radiation oncologists with >6 years experience by referring to the PET and diagnostic CT images.

The database included three tumor types [118 solid, 53 part-solid and 21 pure ground-glass opacity (pure GGO) types]. Solid, part-solid and pure GGO types were defined based on consolidation-to-tumor ratio (CTR), which is calculated by dividing the solid portion size by the total tumor size in a lung window setting. The CTR for solid tumors is one, and that for pure GGO tumors is zero. The part-solid tumors show a CTR between zero and one, but usually around 0.5 [33]. Tumor types were determined by a radiologist (H.Y.) based on the planning CT images at a lung window level and width of −600 and 1500 Hounsfield units (HU), respectively.

### Preprocessing of 3D planning CT images and GTV region datasets

The 3D planning CT and GTV binary images were converted into isovoxel images with a voxel size of 0.977 mm by a shape-based interpolation method [34]. The regions of interest (ROI; 50 × 50 × 6 to 50 × 50 × 72 pixels, hereinafter expressed as 50 × 50 × 6–72 pixels) were cropped based on the centroids of lung cancer regions from the original planning CT images; the dimensions are according to a guideline published in the Executive Summary of an ASTRO Evidence-Based Guideline [35] stating that the cancer regions should be smaller than 50 mm; and the maximum size of lung cancer in the database was 24.716 mm. The cropped ROI images were resized from 50 × 50 × 6–72 pixels to 40 × 40 × 5–58 pixels to meet input image sizes (as a multiple of 8 for the DVNs) in the NiftyNet platform [32].

### Data augmentation

**Fig. 2.**
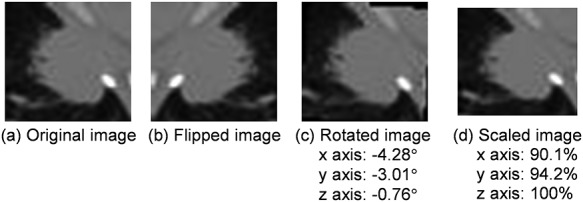
Examples of data augmentation applied in this study: (**a**) an original image, (**b**) flipped image, (**c**) rotated image, and (**d**) scaled image.

Augmentation techniques were used in the training step to avoid deep learning overfitting [36]. The images were randomly rotated from −10° to 10°, randomly flipped, and randomly scaled from −10 to 10%. Figure 2 shows examples of data augmentation applied in this study. Only the training images were augmented; the test images were not.

### DVN segmentation

**Fig. 3.**
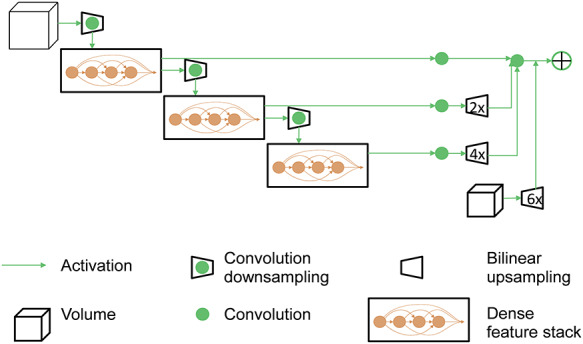
Architecture of dense V-networks used in this study.

Gibson *et al.* [30] proposed DVNs in which the 2D segmentation model of a DenseNet [35] and a 3D medical segmentation model of a V-network [30] are combined for multi-organ segmentations. Figure 3 shows the architectures of the DVNs, which use a fully convolutional neural network based on convolutional units and enable high-resolution activation maps.

DVNs have two main features, the dense connection and V-network. The densely connected network [37] is developed from a network called Residual network (ResNet) [38]. The main idea of dense connection is to input all the features extracted by previous layers, avoiding overfitting when using a deeper network and small dataset [37]. To increase memory efficiency, Gibson *et al*. [30] introduced a new batch-wise spatial dropout that can be combined with those of memory-efficient dense blocks by allocating only shared memory storage for the specific number of computed activation maps. Because dense feature stacks and batch-wise spatial dropouts are memory efficient, deeper networks are enabled, which have the advantage of being able to segment smaller structures [30]. V-network [39] is a network developed from U-network [40]. In V-network, the input images were changed to 3D images and residual learning [38] is employed in U-Net to improve performance. Residual learning has the potential to avoid gradient exploding and vanishing and reduce overfitting, enabling us to build a deeper network for difficult tasks. The dense connections and the V-network structure can significantly improve the performance, making the network more sensitive to small structures [30] and more effective on a small dataset [37]. Therefore, we have used DVNs to segment GTV regions.

Training datasets of the 3D planning CT images and contours of GTVs (reference contours) determined by radiation oncologists were fed into the DVNs as input and annotation data, respectively. Table 1 shows the hyperparameters to be optimized for ranges or candidates. Hyperparameters were optimized in a 10-fold cross-validation test with the 3D-planning CT volumes using a GPU of NVIDIA TITAN X Pascal (NVIDIA Corporation, Los Alamitos, CA).

**Table 1:** Hyperparameters to be optimized for ranges or candidates

Hyperparameters	Range or candidate for optimization					
Loss function	Dice loss [31]	Cross entropy	Generalised Dice overlap [41]	Dice nosquare	Generalised Wasserstein Dice loss [42]	
Learning rate	0.0001	0.001	0.01			
Activation function	Parametric ReLU	selu [43]	Leacky ReLU	ReLU		
Gradient method	RMSprop	momentum	adagrad	Gradient discent	Adam	
Batch size	1	2	4	8	16	32
Iterations	5190	8650	17 300	25 950	86 500	
Dropout	0.75	0.5	0.25			

**Fig. 4.**
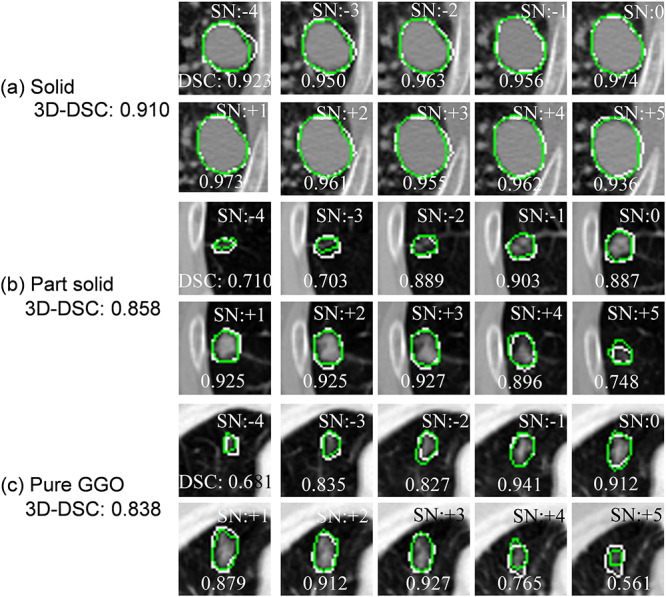
Reference GTVs (white line) and regions estimated with the dense V-networks (green line): (**a**) solid, (**b**) part-solid, and (**c**) pure GGO. 3-D DSCs and slice numbers (SNs) are included in these images. The isocenter slice number is 0.

With three DVNs trained by the same training dataset and the same optimum hyper-parameters but initialed randomly, three output GTVs called DVN1, DVN2 and DVN3 were obtained each time we input a test image. To combine these results, these segmented GTVs produced single outputs, logical AND (common agreement) outputs, and logical OR (summation) outputs by combinations of two or three outputs from the DVNs. Therefore, for the three models DVN1, DVN2 and DVN3, we had four logical AND (AND DVN1–2, AND DVN1–3, AND DVN2–3, AND DVN1–2–3) and four logical OR (OR DVN1–2, OR DVN1–3, OR DVN2–3, OR DVN1–2–3) results. The four logical AND outputs were obtained by combinations of two or three outputs of DVN1, DVN2 or DVN3, and four logical OR outputs using the same combinations as those used in the logical AND outputs.

### Evaluation of GTVs extracted by the proposed approach

The 192 cases were randomly split into 90% training cases and 10% test cases before data augmentation in a 10-fold cross-validation test. In the training step, 85% of the training cases were used for training the DVNs and 15% of the training cases for testing them. Following these settings, the numbers of cases in training, validation and testing datasets were 147, 26 and 19, respectively.

The segmentation accuracy of GTV contours for 192 lung cancer patients (118 solid, 53 part-solid and 21 pure GGO types) were evaluated using a 10-fold cross-validation test using Dice’s similarity coefficients (DSCs) [44] and the Hausdorff distance (HD) [45]. All DSCs and HDs were three-dimensionally derived from 3D volumes generated by DVNs.

The DSC denotes the similarity between the reference region determined by radiation oncologists and the GTV region estimated using the proposed approach; its values range from 0 to 1 and are calculated using the following equation:

(1) \begin{equation*} \mathrm{DSC}=\frac{2n\left(T\cap D\right)}{n(T)+n(D)} \end{equation*}

where *T* is the GTV ground truth region determined by experienced radiation oncologists, *D* is the GTV region estimated using the proposed approach, $n(T)$ is the number of pixels in region $T$, $n(D)$ is the number of pixels in region $D$ and $n(T\cap D)$ is the number of overlapping pixels between $T$ and $D$.

The HD is the degree of misregistration between two sets of data measured by the distance within a point set $A$ that is farthest from any point within a point set $B$ and vice versa [45]. The HD is calculated using the following equation with the two finite point sets $A=\{{\boldsymbol{a}}_1,\dots, {\boldsymbol{a}}_p\}$ and $B=\{{\boldsymbol{b}}_1,\dots, {\boldsymbol{b}}_q\}$(${\boldsymbol{a}}_i$ and ${\boldsymbol{b}}_i$ are defined as the position vectors):

(2) \begin{equation*} H\left(A,B\right)=\mathit{\max}\left(h\left(A,B\right),h\left(B,A\right)\right) \end{equation*}

where

(3) \begin{equation*} h\left(A,B\right)=\underset{{\boldsymbol{a}}_i\in A}{\max}\underset{{\boldsymbol{b}}_i\in B}{\min}\left\Vert{\boldsymbol{a}}_i-{\boldsymbol{b}}_i\right\Vert \end{equation*}

and $\Vert \cdot \Vert$ is some underlying norm describing the points within $A$ and $B$ (e.g. the ${L}_2$ or Euclidean norm).

The HD is the maximum of $h(A,B)$ and $h(B,A)$. The function $h(A,B)$ identifies the point ${\boldsymbol{a}}_i\in \mathrm{A}$ that is farthest from any point of $B$ and measures the distance from ${\boldsymbol{a}}_i$ to its nearest neighbor in $B$ (using the given norm $\Vert \cdot \Vert$). If two images are identical, the score is zero, and the score is larger as the shapes of the two images are more different.

## RESULTS

**Fig. 5.**
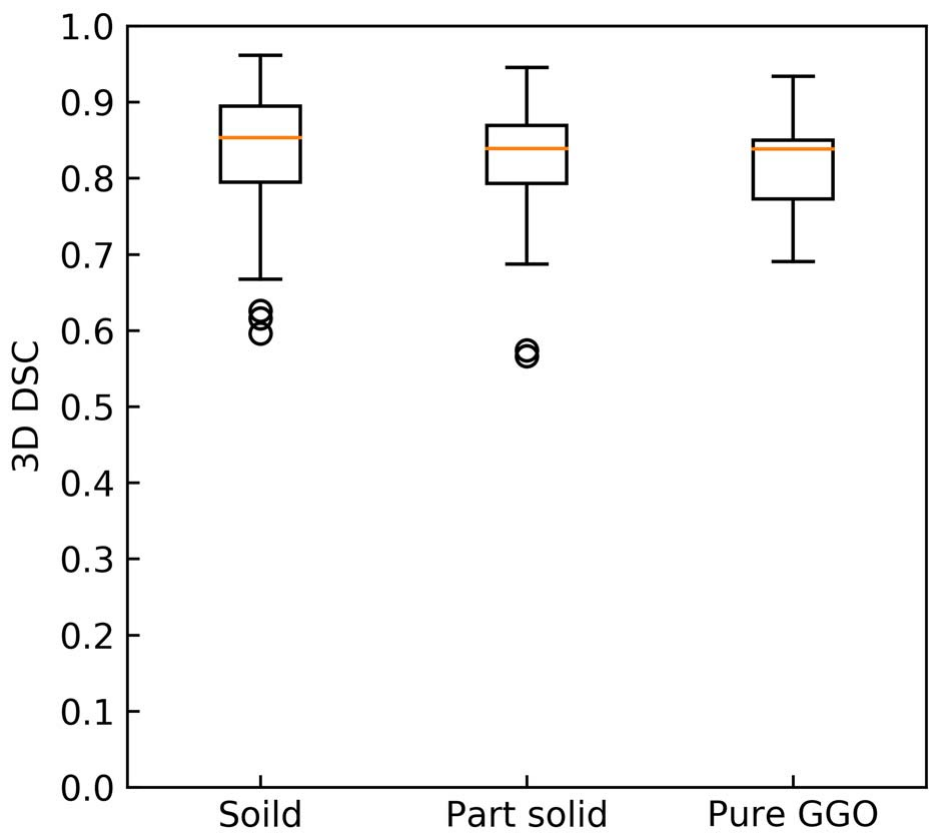
3D-DSCs for solid, part-solid and pure GGO types.

**Fig. 6.**
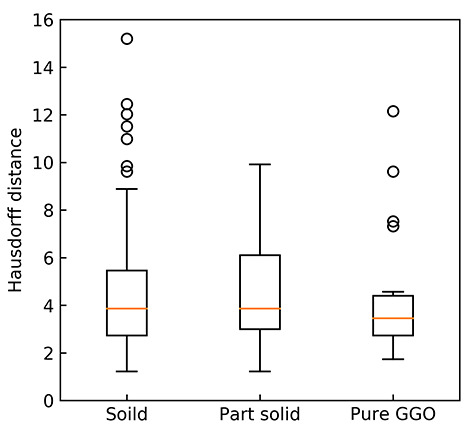
HDs for solid, part-solid and pure GGO types.

Figure 4 illustrates instances of each tumor type that shows reference GTVs (white line) and regions estimated by the proposed approach (green line; using the Dice loss function, a learning rate of 0.001, an activation function of selu, a batch size of 16, a gradient method of RMSprop, a dropout ratio of 0.75 (keep) and an iteration of 8650). The calculation time for delineation of a lung tumor was about 0.2 s on average. Table 2 shows DSCs and Table 3 shows HDs for 11 segmented GTVs obtained from three single outputs, four logical AND outputs, and four logical OR outputs using combinations of two or three outputs from DVNs based on a 10-fold cross-validation test. Figures 5 and 6 show 3D-DSCs and HDs obtained by the AND DNV 1–2–3 for solid, part-solid, and pure GGO tumor types, respectively. The average 3D DSCs for solid, part-solid, and pure GGO (Fig. 5) types were 0.838 ± 0.074, 0.822 ± 0.078, and 0.819 ± 0.059, respectively. The average HDs for solid, part-solid and pure GGO (Fig. 6) types were 4.03 ± 1.94 mm, 4.07 ± 2.02 mm and 4.70 ± 2.61 mm, respectively.

**Table 2:** DSCs and HDs for 11 segmented GTVs obtained from three single outputs, four logical AND outputs, or four logical OR outputs by combinations of two or three outputs from DVNs based on a 10-fold cross validation test

	DVN1	DVN2	DVN3	AND DVN1–2	AND DVN1–3	AND DVN2–3	OR DVN1–2	OR DVN1–3	OR DVN2–3	AND DVN1–2–3	OR DVN1–2–3
DSC	0.822	0.822	0.820	0.831	0.829	0.829	0.814	0.814	0.813	0.832	0.808
HD	4.90	4.85	5.01	4.62	4.60	4.63	5.04	5.21	5.18	4.57	5.28

**Table 3:** DSCs of different types of lung nodule for 11 segmented GTVs obtained from three single outputs, four logical AND outputs, or four logical OR outputs by combinations of two or three outputs from DVNs based on a 10-fold cross validation test

	DVN1	DVN2	DVN3	AND DVN1–2	AND DVN1–3	AND DVN2–3	OR DVN1–2	OR DVN1–3	OR DVN2–3	AND DVN1–2–3	OR DVN1–2–3
All	0.822	0.822	0.820	0.831	0.829	0.829	0.814	0.814	0.813	0.832	0.808
Solid	0.826	0.825	0.825	0.836	0.834	0.835	0.816	0.817	0.816	0.838	0.810
Part solid	0.819	0.819	0.818	0.823	0.822	0.824	0.815	0.816	0.814	0.822	0.810
Pure GGO	0.810	0.814	0.795	0.823	0.815	0.813	0.801	0.790	0.796	0.819	0.788

**Fig. 7.**
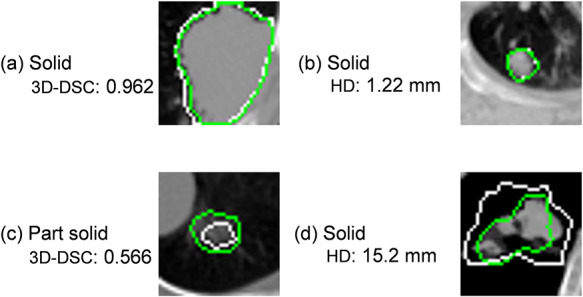
Reference GTVs (white line) and regions estimated with the DVNs (green line): (**a**) highest 3D DSC, (**b**) highest HD, (**c**) lowest 3D DSC, and (**d**) lowest HD.

The highest 3D DSC and lowest HD values obtained from AND DVN1–2–3 were 0.962 and 1.22 mm, respectively, whereas the lowest 3D DSC and highest HD values obtained similarly were 0.566 and 15.2 mm, respectively. These values derive from different cases, and the case with the highest 3D DSC value (Fig. 7a) has a distinct boundary owing to the high contrast between the lung tumor and its surrounding background tissue. On the other hand, the case with most accurate HD (Fig. 7b) is a small and solid tumor with a distinguishable outline. The case with the lowest 3D DSC is one in which the differences between lung cancer and blood vessels are difficult to identify, as shown Fig. 7c. The case with the least accurate HD (Fig. 7d) is a large solid tumor with a blurred contour.

**Fig. 8.**
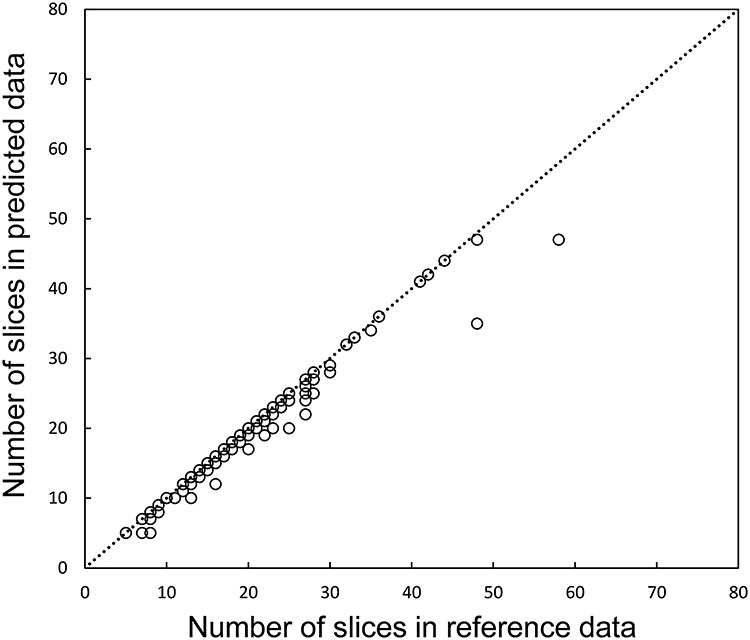
Relationship between the numbers of slices with reference and predicted contours of AND DNV1–2–3.

The relationship between the numbers of slices with reference and predicted contours is shown in Figure 8. In all, 2.9% (112 slices in 75 lung tumors) of 3823 slices in 192 lung tumors were underestimated in terms of the numbers of slices with predicted contours.

## DISCUSSION

Table 4 compares the proposed approach with four automated approaches developed in past studies to delineate GTV regions for lung SBRT or NSCLC patients who have bigger GTVs than SBRT patients. Compared to machine learning-based studies with 2D images [16, 17], the accuracies of the proposed approach were higher for all three types of cancers viewed with 3D images, especially the part-solid and pure GGO types. This is because of the difficulty involved for machine learning to learn heterogeneity in the part-solid and pure GGO types, as shown in Figs 4 b and c. Besides, applying 3D images instead of 2D images can involve more information, e.g. the continuity of tumor region in several slices, making it possible for the segmentation system to learn more features among contiguous slices.

**Table 4:** Comparison of the proposed approach with four automated approaches for delineation of GTV regions for lung SBRT or lung cancer patients

	No. of patients	Modality	Machine learning	Results
Our study	192 (Lung SBRT) Solid: 118 Part solid: 53 Pure GGO:21 10-fold cross validation test (training: 174–175, test: 19–20)	3D-CT	DVNs	DSC: 0.832 HD: 4.57 mm Solid: 0.838 Part solid: 0.822 Pure GGO: 0.819
Kawata *et al*. [16]	16 (Lung SBRT) Solid: 6 Part solid: 6 Pure GGO: 4	2D-PET/CT	Fuzzy-c-means clustering method-based framework	DSC: 0.79 Solid: 0.83 Part solid: 0.76 Pure GGO: 0.79
Ikushima *et al*. [17]	14 (Lung SBRT) Solid: 6 Part solid: 4 Pure GGO: 4	2D-PET/CT	Support vector machine	DSC: 0.777 Solid: 0.834 Part solid: 0.701 Pure GGO: 0.763
Zhong *et al*. [25]	60 (Lung SBRT) Training: 38 Test: 22	3D-PET/CT 3D-CT 3D-PET	DFCN-based cosegmentation (DICELoss) 3D-Unet (DICELoss) 3D-Unet (DICELoss)	DSC: 0.861 (CT) DSC: 0.828 (PET) DSC: 0.811 DSC: 0.794
Zhao *et al*. [27]	84 (Lung cancer) Training: 48 Test: 36	3D-PET/CT 3D-CT 3D-PET	Multi-modality FCN CNN CNN	DSC: 0.85 DSC: 0.76 DSC: 0.83

Our proposed approach failed in segmenting 75 lung tumors in 112 slices of 75 patients. These tumor regions were not segmented in the first, the last and central slices 70, 17 and 25, respectively. Therefore, the numbers of slices with predicted contours were underestimated as shown in Fig. 8. It is still uncertain why DVN failed to segment in those slices. Besides, when we combined three DVN outputs by using a logical AND method, there were more segmentation failures, because we successfully segmented the tumors on slices by AND DVN only if the two (or three) DVN models reach a common agreement. In further research, we will find some other method to avoid the failure that happened in DVNs and logical AND DVNs.

The proposed approach is a deep learning method based on supervised learning, which could not avoid the impact of the variability of the reference data on the performance, since the reference data were subjectively decided by human observers. One choice to reduce the variability is to use an unsupervised learning method, which does not need any reference data for training. However, it is hard to evaluate the results of unsupervised learning due to the lack of reference data (‘truths’). Therefore, further research is needed to develop new methods for reducing the reference variability and evaluating the segmentation accuracy at the same time.

Past studies on deep learning with SBRT or lung cancer patients [25, 27] (Table 4) achieved better performances than the proposed approach with both PET images and CT images. Because applying PET and CT images at the same time may cause a misregistration problem, it increases the difficulty of collecting data and additional work to register the tumor boundaries between PET and CT images; we employed only CT images, which can avoid misregistration on tumor boundaries between PET and CT images. Besides, a bigger database (192 cases in total) is employed than in past study, which reduces the risk of overfitting. Moreover, employing the common agreement (AND region) to delete outliers can increase the level of accuracy over those in past deep learning-based studies using a single modality. This also means that DVNs may increase the segmentation accuracy of GTVs if we used dual modalities (PET/CT images), like past studies.

There are three limitations in this study. First, the number of cases is not sufficient to improve segmentation performances, especially in pure GGO and part-solid tumor types. Second, we focused on GTVs instead of CTVs. Because radiation therapy requires CTVs to determine the appropriate PTVs (by adding internal and set-up margins), segmentation approaches for CTVs should be developed in future work, which we aim to accomplish. Third, we did not have a way to interpret the DSC to evaluate the impact on clinical contouring. Compared to the past study shown in Table 4, there is no significant improvement in the present study (in some cases results are even a little bit worse). But we do not know whether a small change in DSC or HD can affect the clinical contouring or not. The ‘good enough’ DSC is still unclear so we cannot evaluate the impact on clinical use.

## CONCLUSIONS

We developed an automated approach for extracting GTVs of lung cancer patients using datasets of 3D planning CT images to segment tumors by deep learning-based DVNs. The 3D DSC and HD values obtained by the deep learning-based DVNs are 0.832 ± 0.074 and 4.57 ± 2.44 mm (AND DVN1–2–3), respectively. Therefore, the proposed approach has the potential to be useful in delineating GTVs for treatment planning of lung cancer SBRT and to assist radiation oncologists.
